# Mobile RNAs as systemic signaling beyond boundaries for plant stress resistance

**DOI:** 10.3389/fpls.2025.1712714

**Published:** 2026-01-02

**Authors:** Shujin Lin, Haining Li, Shiyan Bai, Xiao Han

**Affiliations:** College of Biological Science and Engineering, Fuzhou University, Fuzhou, China

**Keywords:** mobile RNAs, stress resistance, systemic signaling, RNA trafficking, RNAi applications

## Abstract

Plant mobile RNAs—including small RNAs (miRNAs, siRNAs), mRNAs, lncRNAs, and tRNA fragments—function as systemic signaling molecules that traverse cellular, tissue, and species boundaries to coordinate plant adaptation to environmental stresses. Here, we summarize the critical roles of mobile RNAs in mediating systemic adaptation to abiotic challenges and biotic interactions. Crucially, we highlight the diverse transport mechanisms enabling their movement and discuss the emerging functional versatility of mobile RNAs, which extends beyond transcriptional regulation to encompass epigenetic modifications, resource allocation, and cross-species communication. These fundamental insights are driving transformative applications: Mobile RNAs provide the foundation for developing systemic RNAi-based biopesticides and are being integrated with CRISPR-Cas technologies to overcome delivery barriers and enable heritable, transgene-free genome editing in crops. Understanding and harnessing mobile RNA networks offers unprecedented potential for engineering resilient crops and implementing precise, sustainable crop protection strategies to address global food security challenges.

## 1 Introduction

Within plants, there exists a class of regulatory molecules—mobile RNAs—endowed with the ability to traverse cells, undergo long-distance transport, and even transmit across species. These include small RNAs such as miRNAs/siRNAs, messenger RNAs (mRNAs), long non-coding RNAs (lncRNAs), and tRNA fragments. In the face of a dynamically changing natural environment, mobile RNAs act as key hubs in plant responses to abiotic stresses (such as drought, salinity, and extreme temperatures) and biotic stresses (such as pathogen infection and parasitic plant attachment) through precise molecular regulatory networks. Capable of breaking cellular boundaries at specific times, mobile RNAs function as signaling molecules to transmit environmental response signals between different tissues, organs, or even species. This spatiotemporal dynamic signal transmission capability endows plants with unique strategies for growth, development, and adaptation to diverse adverse conditions (Pagliarani and Gambino, 2019).

A defining feature of plant mobile RNAs lies in their ability to transcend the constraints of their biosynthesis sites, forming regulatory networks that span cells, tissues, and even species. Small RNAs (20–24 nt) can diffuse between adjacent cells via plasmodesmata. More importantly, they can undergo long-distance transport through the vascular system, enabling signal transmission between organs (Song et al., 2021; Ouyang et al., 2024). Certain miRNAs or lncRNAs are transported from buds or leaves to roots or developing leaves to maintain ion homeostasis or participate in chromatin remodeling, coordinating early stress signals at the whole-plant level (Pagliarani and Gambino, 2019; Zhang et al., 2019; Jha et al., 2020). Other miRNAs can be transported upward from roots to shoots in response to belowground environmental changes, thereby regulating gene expression (Yan, 2022). Key developmental regulators, such as the mRNA of *FLOWERING LOCUS T (FT)*—a classic long-distance flowering signal—are transported from leaves to the shoot apical meristem via the phloem, inducing inflorescence morphogenesis (Takagi et al., 2024). Even mRNAs derived from pollen can move from seeds to fruits, influencing fruit ripening and quality (Wang et al., 2024).

Abiotic stresses (e.g., osmotic imbalance caused by drought and ion toxicity from salinity) and biotic stresses (e.g., toxins secreted by fungi and resource plunder by parasitic plants) cause global crop yield losses of 30%–50% annually (Singh et al., 2023). Most previous studies have focused on individual genes or local signaling pathways, but the discovery of mobile RNAs reveals that plant responses to biotic and abiotic stresses require coordinated signal transmission across organs, with mobile RNAs playing an irreplaceable role in systemic regulation. For instance, certain sRNAs and lncRNAs can move between organs to execute critical regulatory functions in response to various environmental stresses (Singh et al., 2023). Additionally, some small RNAs and mRNAs can achieve cross-species transmission via exosomes, mediating RNA interactions between plants and pathogens or parasitic plants (Song et al., 2021). This mobility of RNA molecules breaks the spatial constraints of traditional gene regulation, forming complex regulatory networks that serve as core hubs for integrating local and systemic stress responses in plants (Yan, 2022).

In this review, we systematically summarize the core functions of mobile RNAs in plant stress resistance from two perspectives: abiotic and biotic stresses. We also dissect their transport mechanisms and modes of action in depth. Furthermore, we discuss current research challenges and future application prospects—such as intelligent stress-resistant molecular breeding and the development of RNAi-based biopesticides—by integrating cutting-edge technologies like single-cell sequencing and nanodelivery systems. This work aims to provide a solid theoretical foundation and technical support for crop stress resistance improvement under global climate change.

## 2 Stress-resistance functions of plant mobile RNAs

**Figure 1:**
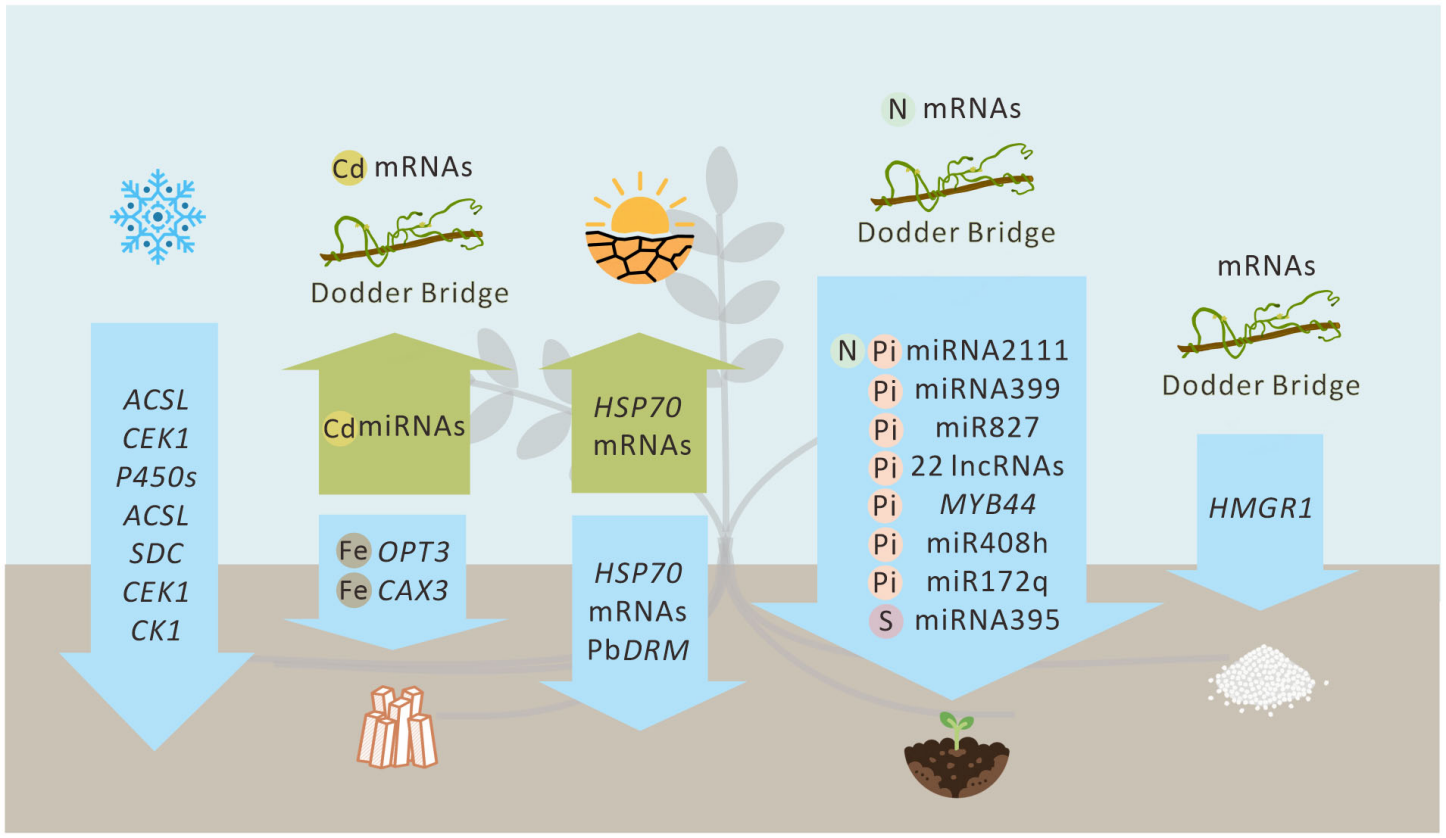
Transport of mobile RNAs under abiotic stress via vascular tissues and parasitic plants. Abiotic stressors depicted (from left to right): chilling stress, heavy metals, drought, nutrient deficiency, and salinity. Blue arrows denote phloem transport. Green arrows denote xylem transport. Transport mediated by the parasitic plant *Cuscuta* spp. (dodder bridge) is indicated.

Plant mobile RNAs exhibit highly specific regulatory functions in responding to abiotic (**Figure 1**, **Table 1**) and biotic stresses. They construct efficient systemic signal transduction networks via the vascular system, forming critical “perception–execution” regulatory loops that serve as core molecular bridges connecting local stress signals to global adaptive responses.

**Table 1:** Mobile RNAs involved in plant responses to abiotic stresses with transport pathways.

Stress category	Abiotic stress type	Mobile RNA type	Species	Transport pathway	PMID
Extreme temperature	Chilling stress	*CsaACSL, CsaCEK1, CsaP450s* etc.	*Cucumis sativus*	Phloem	35184197
	Chilling stress	*CmoASCL, CmoSDC, CmoCEK1* etc.	*Cucurbita moschata*	Phloem	35184197
	Chilling stress	*CmoCK1*	*Cucurbita moschata*	Phloem	39325727
Drought	Drought response	*CmHSP70* (Heat Shock Protein 70)	*Cucurbita moschata*	Phloem and xylem	35163839
	Drought response	mRNAs	*Pyrus betulaefolia*	Phloem	32081266
	Drought response	*PbDRM* (DROUGHT-RESPONSIVE MOBILE GENE)	*Pyrus betulaefolia Bunge*	Phloem	32081266
	Drought response	mRNAs	*Solanum pennellii*	Phloem and xylem	40243940
Heavy metal stress	Cadmium Stress	mRNAs	*soybean*	Dodder bridge	40313728
	Cadmium Stress	miRNAs	*Zea mays L.*	Xylem	30909604
	Fe deficiency	*MdOPT3*	*Malus domestica/Malus baccata*	Phloem	34618059
	Fe deficiency	*MdCAX3*	*Malus domestica*	Phloem	35254714
Nutrient deficiency	Nitrate (N) deficiency	mRNAs	*Cucumis sativus*	Dodder bridge	33793912
	Nitrate (N) deficiency	miRNA2111	*Lotus japonicus*	Phloem	38057302
	Phosphate (Pi) deficiency	miRNA399	*Arabidopsis thaliana/Brassica napus/Cucurbita maxima*	Phloem	17988220
	Phosphate (Pi) deficiency	miR399d、miR399d*	*Arabidopsis thaliana*	Phloem	28322489
	Phosphate (Pi) deficiency	miR827	*Arabidopsis thaliana*	Phloem	28322489
	Phosphate (Pi) deficiency	miR2111a	*Arabidopsis thaliana*	Phloem	28322489
	Phosphate (Pi) deficiency	22 lncRNAs	*Cucumis sativus*	Phloem	30171742
	Phosphate (Pi) deficiency	*AtMYB44*	*Arabidopsis thaliana*	Phloem	37896080
	Phosphate (Pi) stress	CsmiR399a, CsmiR399e, CsmiR408h, CsmiR172q	*Cucumis sativus*	Phloem	27249565
	Sulfate (S) deficiency	miRNA395	*Arabidopsis thaliana*	Phloem	20388194
Salt tolerance	salinity stress	mRNAs	*Citrus sinensis*	Dodder bridge	36046588
	salinity stress	mRNAs	*Cucumis sativus*	Dodder bridge	31665509
	salinity stress	*PbHMGR1*	*Pyrus betulaefolia*	Phloem	37059127

### 2.1 Mobile RNA regulation in abiotic stress responses

#### 2.1.1 Drought

Drought induces osmotic imbalance in plant cells, and mobile RNAs mediate adaptive responses by regulating root development, plasma membrane transporters, and antioxidant systems. Through complex and diverse mechanisms, mobile RNAs participate in transcriptional regulation and signal transduction during drought stress. Using RNA sequencing (RNA-seq) combined with SNP analysis, researchers identified 61 upwardly mobile and 990 downwardly mobile mRNAs in tomato heterografts; these mRNAs were functionally enriched in RNA binding, photosynthesis, and heat response, demonstrating that mobile mRNAs enhance drought tolerance by regulating gene expression (Du et al., 2025). Similarly, RNA-seq analyses in Pyrus species revealed drought-responsive mobile mRNAs. Transport of the mobile mRNA *PbDRM* (*Pyrus betulaefolia* drought-responsive mobile mRNA) from the rootstock *Pyrus betulaefolia Bunge* to multiple scion varieties significantly increases under drought (Wang et al., 2020). Knockdown of *PbDRM* enhances drought sensitivity in *P. betulaefolia*, indicating its key role in improving overall plant drought tolerance.

Upon drought perception in roots, systemic signaling molecules—including mobile RNAs—may coordinate responses in aerial tissues such as stomatal closure and osmolyte biosynthesis. Among known mobile miRNAs, miR399 serves as a classic example of a long-distance signal that moves from shoot to root to regulate phosphate homeostasis (Buhtz et al., 2010; Chiang et al., 2023). Interestingly, miR399 has also been reported to modulate stomatal development through the PHO2 pathway (Zhu et al., 2020), suggesting that certain mobile miRNAs may integrate environmental cues perceived in roots with stomatal responses in leaves. While direct evidence for drought-induced miR399 mobility remains limited, this example highlights a plausible mechanism by which mobile small RNAs could participate in coordinating water stress responses between roots and shoots. Concurrently, mature leaves may transport drought-responsive mobile RNAs through phloem sieve tubes to roots, as some RNAs potentially remodel root development programs (Zhang et al., 2021).

Notably, the enrichment of heat-responsive genes is of great significance. Drought is often accompanied by high-temperature stress, and systemic RNA signaling must be considered in the context of combined stress responses. Heat-responsive programs (e.g., induction of heat-shock proteins and antioxidant pathways) contribute to longer-term cellular protection and can interact with drought-response networks (Sato et al., 2024); thus, elucidating how shoot-to-root and root-to-shoot RNA fluxes integrate with heat- and drought-regulated transcriptional modules will be important for understanding whole-plant acclimation under compound stress.

#### 2.1.2 Salt stress

In plant responses to salt stress, mobile RNAs participate in the stress response process through two distinct mechanisms: triggering transcriptome changes and acting as directly transported signaling molecules. In citrus, for example, by constructing a community of Hamlin sweet orange plants connected via *Cuscuta* (dodder), researchers found that vascular system signals induced by salt stress can be transmitted through *Cuscuta* to interconnected host plants, eliciting transcriptional responses to salt stress in the hosts. The *Cuscuta*-mediated vascular signals activate stress responses in citrus leaves, leading to transcriptome reconfiguration; meanwhile, systemic signals significantly regulate secondary metabolic pathways associated with abiotic stress, including those involved in the metabolism of phenylpropanoids, lignins, and lignans (Duan et al., 2022). Additionally, in pear trees, the mRNA encoding 3-hydroxy-3-methylglutaryl-CoA reductase 1 (*PbHMGR1*) undergoes long-distance transport via the phloem. Tobacco plants overexpressing this mRNA exhibit enhanced salt tolerance during seed germination, and increased relative abundance of *PbHMGR1* in heterografted scions enables plants to avoid severe damage under salt stress (Hao et al., 2023).

A core challenge of salt stress lies in ionic toxicity—such as high Na^+^ concentrations—perceived by the roots. Directional transport of substances within the vascular system is critical for mobile RNA-mediated systemic salt tolerance. Upon perceiving salt stress, roots synthesize specific mobile RNA signals (e.g., miRNAs responsive to ionic imbalance), which are rapidly transported upward to aerial parts via the transpiration stream in xylem vessels. This triggers protective responses in organs like leaves, including ion sequestration (e.g., compartmentalization of Na^+^ into vacuoles), activation of antioxidant defenses, and adjustment of photosynthetic efficiency (Prince et al., 2017; Yang et al., 2022). Concurrently, mobile RNAs synthesized in aerial parts—such as the aforementioned PbHMGR1 mRNA or miRNAs regulating Na^+^/H^+^ antiporters—are actively transported to the roots via phloem sieve tubes. These mobile RNAs function in the roots to enhance ion efflux capacity of root cells, maintain membrane integrity, and regulate root architecture to optimize selective ion uptake (e.g., K^+^ vs. Na^+^). However, comprehensive research on the specific functional networks of mobile RNAs in regulating selective ion uptake in roots and their systemic integration mechanisms remains lacking, and the relevant mechanisms have not yet been systematically elucidated.

#### 2.1.3 Extreme temperature stress

Among extreme temperature stresses, chilling stress causes particularly significant damage to plants, and mobile RNAs respond through a variety of metabolic regulatory mechanisms. In cucurbit grafting systems (e.g., watermelon/bottle gourd and cucumber/pumpkin), cold stress triggers a bidirectional mRNA mobility network: mRNAs from pumpkin rootstocks enhance the cold tolerance of scions by regulating fatty acid β-oxidation and amino acid metabolism, whereas mRNAs from cucumber scions are transported back to rootstocks through the regulation of sulfur metabolism and circadian rhythms, forming a synergistic response mechanism (Wang et al., 2020; Liu et al., 2022; Lang et al., 2024). In cucumber/pumpkin grafts, studies have found that 70% of mobile mRNAs show a downward movement direction. Among them, 1,739 mRNAs derived from cucumber scions enhance cold tolerance by regulating sulfur metabolism and circadian rhythms, and 89 mRNAs derived from pumpkins mediate the pathways of 2-oxocarboxylic acid and amino acid synthesis (Liu et al., 2022). Their directional mobility has been verified by RT-PCR of genes such as *CmoASCL* and *CsaACSL*, as well as experiments involving the exogenous addition of glycerophospholipid metabolites (PC/PE). Recent studies have revealed that the cold-induced mobility of pumpkin choline kinase 1 (CK1) mRNA depends on epitranscriptomic regulation via m^5^C and m^6^A modifications (Li et al., 2025). Additionally, the identification of 544 seasonally dynamic mobile mRNAs in apple grafting systems further indicates that the mechanisms of cold-responsive RNA transport are species-specific (Lee et al., 2023). Notably, the mobility mechanism of pumpkin CK1 mRNA differs from the tRNA-like sequence (TLS)-mediated transport pathway of AtCK1 in Arabidopsis, suggesting the complex diversity of cold-responsive RNA transport.

Mobile RNAs achieve this through the integration of membrane stability maintenance, metabolic pathway reprogramming, and epigenetic modification regulation. From a systemic adaptability perspective, although aboveground tissues directly perceive temperature changes, the mobile RNAs they generate may be transported downward to the root system via the phloem, driving whole-plant stress-resistant reorganization. On one hand, systemic signals can reshape root architecture (for example, by reducing non-essential lateral root formation or by promoting lateral root initiation in response to resource cues), thereby improving the plant’s capacity to forage for water. On the other hand, it redistributes photosynthetic products, prioritizing energy supply to meristems and stressed leaves while downregulating energy-consuming metabolic pathways in roots. This enables the optimal allocation of carbon assimilates and nutrient resources throughout the plant, ultimately establishing an integrated functional organism that synergistically resists environmental stress.

#### 2.1.4 Nutritional stress

Plant growth and development depend on the balanced supply of multiple nutrients. However, in natural environments, conditions such as soil nutrient deficiency and imbalance frequently induce nutrient stress, impeding normal plant growth and affecting crop yield and quality. Various mobile miRNAs, lncRNAs, and mRNAs have been detected in plant phloem and are found to act as long-distance signals extensively involved in plant responses to nutrient stress. For instance, under phosphorus stress, *AtMYB44* mRNA is transported from shoots to roots, where it negatively regulates the phosphate transporters PHT1;2 and PHT1;4. Knockout mutants of this gene exhibit enhanced phosphate uptake and increased root biomass (Olukayode et al., 2023). Additionally, studies indicate that lncRNAs may participate in the early response to phosphorus stress by regulating miRNA activity. In pumpkin, phosphorus starvation-induced miR399 moves from aboveground tissues to roots via the phloem, silencing the root PHO2 gene to regulate phosphate uptake and transport—this marked the first demonstration of the systemic function of mobile miRNAs (Pant et al., 2008). In cucumber under phosphorus deficiency, lncRNAs, miRNAs, and mRNAs are enriched in the phloem and systemically transported to root tips and young leaves. Mobile lncRNAs containing CU-rich motifs coordinate phosphorus stress signals (Zhang et al., 2019). The phloem transport of these mRNAs exhibits sink tissue-targeting specificity (such as root tips, shoot apices, and developing leaves), and even those with low abundance in the phloem can undergo long-distance transport (Zhang et al., 2016). Furthermore, the miR399f/miR399f* duplex is unloaded in a dose-dependent manner through root phloem parenchyma cells (PPP), mediating long-distance silencing signals to coordinate phosphate homeostasis (Chiang et al., 2023).

Under iron stress, in apple, *MdCAX3* mRNA is transported from leaves to roots. Upon activation by *MdCXIP1*, it regulates root apoplastic pH and zinc vacuolar compartmentalization, responding to iron starvation to balance iron-zinc homeostasis (Hao et al., 2022). Under cadmium stress, differentially expressed miRNAs in maize xylem sap, including 10 responsive miRNAs, may participate in metal handling, stress responses, and cellular signal transduction by cleaving target genes (Wang et al., 2019). Recent studies have shown that under cadmium stress, the parasitic plant dodder can transport mobile mRNAs through dodder bridges, directly or indirectly inducing transcriptomic changes in donor plants and neighboring recipient plants. These changes involve pathways such as plant–pathogen interactions and phenylpropane biosynthesis and trigger Ca^2+^ and ROS signaling (Pan et al., 2025).

Mobile RNAs are key mediators of interorgan communication in plants, coordinating nutrient acquisition, allocation, and associated physiological and developmental adaptations. They convert locally perceived environmental signals (e.g., specific nutrient deficiencies) into transmissible molecular instructions, relaying them to distal roots or leaves to trigger appropriate responses—such as inducing transporter expression, altering root architecture, or modulating metabolic pathways. This systemic coordination enables plants to function as integrated units, efficiently adapting to complex and fluctuating environmental stresses, which is fundamental for survival and reproduction. Despite the identification of numerous mobile RNAs functioning under nutrient stress, key questions remain unresolved. These include the mechanisms underlying their precise target recognition and potential synergistic or antagonistic interactions between different types of mobile RNAs during nutrient stress responses.

### 2.2 Mobile RNAs in biotic stress responses

In the ongoing coevolutionary arms race between plants and pathogens spanning hundreds of millions of years, mobile RNAs have emerged as key mediators. They establish systemic immune networks spanning organs and tissues via long-distance signaling and act as critical regulators reprogramming host development and nutrient metabolism. Plants deploy defensive RNAs to suppress pathogen invasion while reallocating resources for survival; conversely, during mutualistic symbioses, mobile RNA signals facilitate nutrient exchange and developmental coordination (**Table 2**).

**Table 2:** Characteristics of mobile RNAs involved in organism-plant interactions.

Organism type	Type of mobile RNA	Direction of movement	Organism species	Plant species	Target gene/function	References
Pathogenic	Bc-siR37	Pathogen → Plant	*Botrytis cinerea*	*Arabidopsis thaliana*	Defense-related genes such as *WRKY7, WRKY57, FEI2* (*LRR-RK*), *PMR6*, and *ATG5*	28267415
	Bc-siR3.1	Pathogen → Plant	*Botrytis cinerea*	*Arabidopsis thaliana*	*PRXIIF*, involved in oxidative stress response	24092744
	Bc-siR3.2	Pathogen → Plant	*Botrytis cinerea*	*Arabidopsis thaliana*	*MPK1*, *MPK2*, involved in immune signal transduction	24092744
	Bc-siR5	Pathogen → Plant	*Botrytis cinerea*	*Arabidopsis thaliana*	*WAK*, involved in cell wall defense and immune signaling	24092744
	Bc-siR3.2	Pathogen → Plant	*Botrytis cinerea*	*Solanum lycopersicum*	*MAPKKK4* (Mitogen-Activated Protein Kinase Kinase Kinase)	24092744
	TAS1c-siR483	Plant → pathogen	*Botrytis cinerea*	*Arabidopsis thaliana*	*Vps51* (Vacuolar Protein Sorting 51), *DCTN1* (Dynactin Activator Subunit), SAC1-like Phosphoinositide Phosphatase, etc.	29773668
	TAS2c-siR453	Plant → Pathogen	*Botrytis cinerea*	*Arabidopsis thaliana*	*Vps51* (Vacuolar Protein Sorting 51), *DCTN1* (Dynactin Activator Subunit), SAC1-like Phosphoinositide Phosphatase, etc.	29773668
	mRNA	Plant → Pathogen	*Botrytis cinerea*	*Arabidopsis thaliana*	*SAG21, APS1, PRXIIC, HEL*, etc.	38103543
	BABA-induced 24nt sRNAs	Plant vascular system	*Botrytis cinerea*	*Solanum lycopersicum*	COPII-coated vesicle budding; Aspartate family amino acid biosynthetic processes; Vesicle budding from membrane; Aspartate family amino acid metabolic processes	39562729
	Y-sat derived siRNA	Pathogen → Plant	*Cucumber mosaic virus (CMV)*	*Nicotiana tabacum、Nicotiana benthamiana、Arabidopsis thaliana、Solanum lycopersicum*	*ChlI* (Magnesium Protoporphyrin Chelatase Subunit I)	21573143
	Fol-milR1	Pathogen → Plant	*Fusarium oxysporum f.sp. lycopersici*	*Solanum lycopersicum*	*FRG4* (Calcineurin B-like Interacting Protein Kinase), inhibiting host defense signals	33960431
	sRNA (21 nt)	Pathogen → Plant	*Hyaloperonospora arabidopsis*	*Arabidopsis thaliana*	WNK2 (Kinase), AED3 (Protease)	32441255
	sRNA (19–23 nt, including miRNA-like RNA, phasiRNA)	Pathogen → Plant	*Puccinia striiformis f.sp. tritici*	*Triticum aestivum*	NLR; Glutathione S-transferase; Defense-related transcription factor genes such as bZIP	36427746
	sRNA (20–24 nt)	Plant → Pathogen	*Puccinia striiformis f.sp. tritici*	*Triticum aestivum*	Glycosyl hydrolase family 26 genes; Ribosomal protein genes; TIMELESS domain protein genes	36427746
	miRNAs	Plant → Pathogen	*Puccinia striiformis f.sp. tritici*	*Triticum aestivum*	Possible effector protein-encoding genes	31998329
	TE-derived sRNA (22–23 nt)	Pathogen → Plant	*Sclerotinia sclerotiorum*	*Arabidopsis thaliana*	*SNAK2* (SNF1-related kinase), *SERK2* (Somatic Embryogenesis Receptor Kinase 2)	31361080
	Vm-milR1	Pathogen → Plant	*Valsa mali*	*Malus x domestica*	*RLKT1*, *RLKT2* (Defense signal-related receptor-like protein kinases)	34981514
	rRNA-derived sRNA (24 nt)	Pathogen → Plant	*Verticillium dahliae*	*Arabidopsis thaliana*	Precursor of MIR157d, indirectly regulating *SPL13A* and *SPL13B*, affecting plant flowering transition	35519822
	miR166	Plant → Pathogen	*Verticillium dahliae*	*Gossypium hirsutum/Arabidopsis thaliana*	*Clp-1* (Calcium-dependent cysteine protease)	35290117
	miR159	Plant → Pathogen	*Verticillium dahliae*	*Gossypium hirsutum/Arabidopsis thaliana*	*HiC-15* (Triterpene C-15 hydroxylase)	35290117
	Xosr001	Pathogen → Plant	*Xanthomonas oryzae pv. oryzicola*	*Oryza sativa*	*JMT1* (Jasmonic acid methyltransferase), inhibiting host jasmonic acid signaling pathway	38909609
Parasitic	lncRNA	Parasite → Plant	*Cuscuta australis*	*Glycine max*	Genes related to “metabolic processes,” “catalytic activity,” “signal transduction,” and “stress response”	35008986
	lncRNA	Plant → Parasite	*Cuscuta australis*	*Glycine max*	Affecting organelle-related functions of dodder	35008986
	miR12463b	Parasite → Plant	*Cuscuta campestris*	*Arabidopsis thaliana*	*BIK1* (5′ region)	29300014
	miR12495	Parasite → Plant	*Cuscuta campestris*	*Arabidopsis thaliana*	*BIK1* (3′ region)	29300014
	miR12480	Parasite → Plant	*Cuscuta campestris*	*Arabidopsis thaliana*	*SEOR1*	29300014
	miR12497a	Parasite → Plant	*Cuscuta campestris*	*Arabidopsis thaliana*	*TIR1*	29300014
	miR12497b	Parasite → Plant	*Cuscuta campestris*	*Arabidopsis thaliana*	*AFB3*, *ABF2*	29300014
	miR12494b	Parasite → Plant	*Cuscuta campestris*	*Arabidopsis thaliana*	*HSFB4/SCZ*	29300014
	sRNA	Parasite → Plant	*Cuscuta campestris*	*Cucumis sativus*	Host mRNAs, possibly involved in regulating host physiological processes	31665509
	CcsRNA0641	Parasite → Plant	*Cuscuta campestris*	*Arabidopsis thaliana*	Disease resistance protein family genes and sRNA-degrading nuclease genes, possibly involved in regulating host defense-related processes	34393597
	CcsRNA4295	Parasite → Plant	*Cuscuta campestris*	*Arabidopsis thaliana*	Golgi nucleotide sugar transporter gene and ARF-GAP domain 6 gene, whose long-distance movement may depend on Arabidopsis SGS3 protein	34393597
	AtsRNA3031	Plant → Parasite	*Cuscuta campestris*	*Arabidopsis thaliana*	Metallopeptidase M24 family protein gene and RNA-binding family protein gene, possibly involved in regulating related physiological processes of parasitic plants	34393597
	AtsRNA4348	Plant → Parasite	*Cuscuta campestris*	*Arabidopsis thaliana*	Multiple leucine-rich repeat protein kinase family genes, possibly affecting the growth or parasitic ability of parasitic plants	34393597
	mRNA and sRNA	Parasite → Plant	*Cuscuta chinensis*	*Citrus sinensis*	Activating transcriptional salt stress response	36046588
	mRNA	Plant → Parasite	*Cuscuta pentagona*	*Arabidopsis thaliana/Solanum lycopersicum*	–	25124438
	siRNAs (21 nt, including 22 nt and 24 nt)	Plant → Parasite	*Cuscuta pentagona*	*Nicotiana tabacum*	*STM* gene	32868415
Mutualistic	tRFs (21 nt, Bj-tRF001/002/003)	Mutualistic organism → Plant	*Bradyrhizobium japonicum*	*Glycine max*	*RHD3a/RHD3b* (Root hair defective 3), *HAM4a/HAM4b* (hairy meristem 4), *LRX5* (Leucine-rich repeat extensin-like 5), regulating nodule formation	31346137
	sRNA (21 nt, including those derived from fungal viruses)	Mutualistic organism → Plant	*Gigaspora margarita*	*Medicago truncatula*	Chitinase, expansin, CCR4-NOT transcription complex protein, etc., possibly involved in symbiosis regulation	32231650
	Pmic_miR-8	Mutualistic organism → Plant	*Pisolithus microcarpus*	*Eucalyptus grandis*	Eucgr.E03170 (CC-NLR, Nucleotide-binding leucine-rich repeat protein), maintaining mycorrhizal symbiosis	35012977
	miRNAs (19–22 nt)	Plant → Mutualistic organism	*Populus trichocarpa*	*Laccaria bicolor/Rhizophagus irregularis*	*TRAPP* (Transport protein particle complex subunit), Kinesin light chain, HMG-box transcription factor, etc.	30936859
	mRNA	Plant vascular system	*Rhizobia*	*Soybean*	Symbiosis-related genes	32221813
	tRFs (21 nt)	Mutualistic organism → Plant	*Rhizobium etli*	*Phaseolus vulgaris*	*RGA1* (GRAS family transcription factor), *AP2* (APETALA-2-like transcription factor)	31346137
	tRFs (21 nt, Glu-TTC, Met-CAT2, etc.)	Mutualistic organism → Plant	*Rhizobium tropici*	*Phaseolus vulgaris*	*ERD* (Early responsive to dehydration protein), *ENOD17* (Early nodulin-like protein 17), *HEMA1* (Glutamyl-tRNA reductase)	31346137
	sRNA	Mutualistic organism → Plant	*Rhizophagus irregularis*	*Medicago truncatula*	*DREPP* (Plasma membrane protein), *NPC4* (Non-specific phospholipase C), *VAPYRIN* (AM symbiosis-specific ankyrin repeat protein), *NB-LRR*, etc.	32231650
	Rir2216	Mutualistic organism → Plant	*Rhizophagus irregularis*	*Medicago truncatula*	*MtWRKY69*	39555692

#### 2.2.1 Plant–fungal interactions

Plant–fungal interactions represent a highly precise and dynamically evolving biological process. Upon fungal infection, plants mobilize mobile RNAs to establish systemic early-warning and defense systems. For example, cotton plants challenged by *Verticillium dahliae* secrete miR159 and miR166 via extracellular vesicles (EVs). These miRNAs precisely target fungal virulence genes, such as *Clp-1* (encoding a Ca^2+^-dependent cysteine protease) and *HiC-15* (encoding a trichodermin C-15 hydroxylase), effectively inhibiting pathogen infectivity and reducing plant damage (Zhu et al., 2022).

In tomato, 24-nt mobile small RNAs are detected during BABA-induced long-lasting defense priming against *Botrytis cinerea*. These RNAs correlate with enhanced expression of induced resistance genes. They mediate the maintenance of resistance signals via the RNA-directed DNA methylation (RdDM) pathway, influencing DNA methylation and gene expression. Such small RNAs move systemically from BABA-treated tissues (e.g., roots or leaves) to untreated distal tissues (e.g., scions or fruits), ensuring coordinated and persistent activation of resistance responses throughout the plant. Their accumulation correlates with elevated expression of defense-related genes, suggesting that mobile small RNAs not only act as signals but also may directly modulate transcriptional defense outputs (Stevens et al., 2025).

Conversely, pathogenic fungi secrete mobile RNAs as effector molecules that systemically suppress plant innate immunity by targeting immune signaling pathways, thereby breaching host defenses. *Botrytis cinerea* secretes sRNAs (e.g., Bc-siR3.1, Bc-siR3.2, and Bc-siR5) that enter plant cells, co-opt Arabidopsis AGO1 protein, and silence host immune genes—including MAPK cascade components and cell wall-associated kinases (*WAKs*)—suppressing immune responses and facilitating fungal infection (Weiberg et al., 2013). He et al. demonstrated that Botrytis cinerea delivers extracellular vesicle (EV)-packed sRNAs (Bc-sRNAs) into Arabidopsis cells, exploiting host AGO1 to silence immunity genes and promote infection. This mechanism involves clathrin-mediated endocytosis (CME), with key fungal molecules (BcPLS1) functioning within EVs (He et al., 2023). Furthermore, Fusarium oxysporum f. sp. lycopersici secretes Fol-milR1, which binds tomato AGO4a protein to silence the host calcium-binding protein kinase gene SlyFRG4, enhancing virulence (Ji et al., 2021). Such effector RNAs diffuse through the vasculature, disrupting immune signaling in distal tissues and achieving whole-plant immunosuppression (Atabekova et al., 2023).

#### 2.2.2 Plant–virus interactions

Mobile RNAs mediate both antiviral defenses and viral counter-defense mechanisms during plant–virus interactions. While sRNAs within Arabidopsis extracellular vesicles (EVs) have been shown to enter bacteria and reprogram their gene expression, their specific role against viruses remains unclear. Crucially, viruses encode various suppressors of RNA silencing (VSRs) to counteract plant immunity. For instance, the Cucumber mosaic virus (CMV) 2b protein binds host AGO1 protein, preventing sRNA loading and thereby disrupting RNAi-based defense (Zhang et al., 2006; Crawshaw et al., 2024).

Virus-derived siRNAs (vsiRNAs) themselves can function as mobile signals, modulating host gene expression. Faced with limited resources, plants must prioritize defense over growth through carbon reallocation. A striking example involves the Y-satellite RNA of CMV: Its derived siRNA targets the chlorophyll biosynthesis gene CHLI in tobacco, inducing leaf yellowing to reduce photosynthetic efficiency and conserve energy. Concurrently, it induces the sucrose transporter SUC2, directing sugars preferentially to infection sites to fuel antiviral mechanisms (Shimura et al., 2011). By exploiting host RNA transport and silencing-suppression pathways, viruses can facilitate the systemic movement of virus-derived small RNAs (vsiRNAs), thereby contributing to wide-ranging modulation of host physiology (Pitzalis et al., 2020; Leonetti et al., 2021; Atabekova et al., 2023; Elvira-Gonzalez et al., 2025). Moreover, mobile virus-derived siRNAs can induce DNA methylation via the RNA-directed DNA methylation (RdDM) pathway, potentially conferring transgenerational effects on resistance. Studies on DNA viruses such as geminiviruses have provided strong evidence that virus-derived siRNAs can guide RNA-directed DNA methylation (RdDM) of viral and host loci (Rodríguez-Negrete et al., 2009; Pooggin, 2013; Ceniceros-Ojeda et al., 2016). During geminiviral replication, the formation of double-stranded DNA intermediates and minichromosomes renders the viral genome a direct target of the host RdDM machinery. For instance, the resistance gene in tomato enhances the production of 24-nt vsiRNAs and promotes hypermethylation of the viral DNA, thereby silencing viral transcription and conferring effective immunity (Voorburg et al., 2021) Furthermore, the physical encapsulation of methylated viral DNA into transcriptionally silent minichromosomes during recovery provides a clear mechanistic link between vsiRNA-guided RdDM and durable virus resistance (Ceniceros-Ojeda et al., 2016). While the mobility of siRNAs through the vasculature to seeds is mechanistically plausible, experimental validation of this phenomenon awaits further investigation.

#### 2.2.3 Parasitic plants

**Figure 2:**
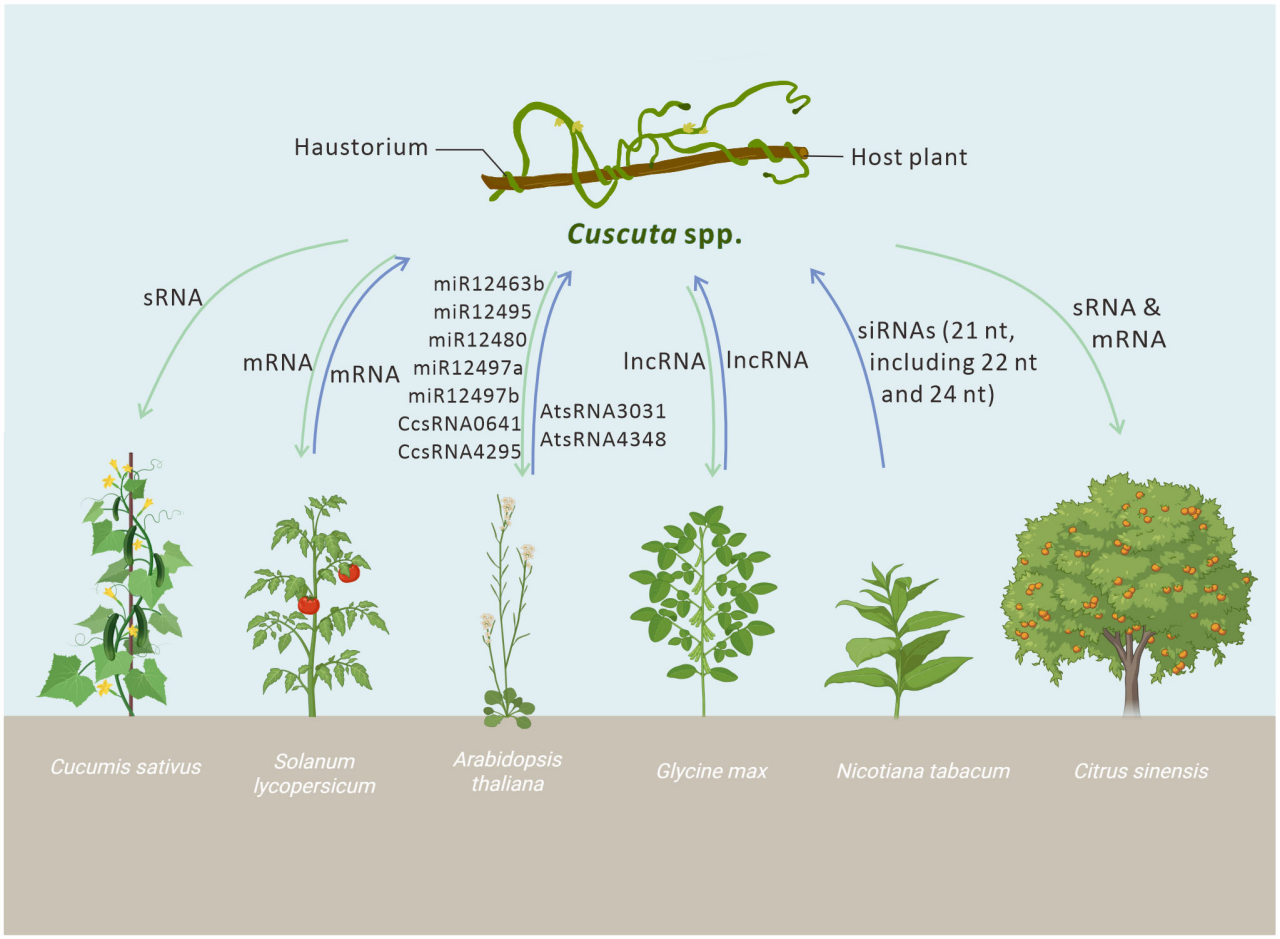
Schematic representation of mobile RNA exchange between the parasitic plant *Cuscuta* spp. (dodder) and its host plant. Blue arrows denote phloem-mediated transport. Green arrows denote xylem-mediated transport. Bidirectional movement of mobile RNAs facilitates communication across the parasitic interface.

A complex bidirectional RNA communication network exists between dodder (*Cuscuta* spp.) and host plants. Dodder establishes intimate connections with hosts via haustoria, enabling the bidirectional transfer of numerous mRNAs, lncRNAs, and small RNAs (sRNAs, including miRNAs and siRNAs) across species (**Figure 2**) (Kim et al., 2014; Subhankar et al., 2021; Wu et al., 2022).

Host plant mRNAs can be transferred across species to dodder through haustoria, where they are translated and exert functions (Park et al., 2021). For instance, the florigen FT mRNA from tobacco (*Nicotiana benthamiana*) is transferred to dodder (*C. australis*) and induces flowering in dodder itself (Shen et al., 2020). In terms of parasitic suppression, defensive sRNAs (such as siRNAs/miRNAs) produced by host plants can be transported over long distances to dodder via phloem, triggering cross-species gene silencing (Subhankar et al., 2021). Representative cases include host-derived sRNAs targeting the *SHOOT MERISTEMLESS-like (STM)* gene in dodder, which interferes with the development of its apical meristem and thereby inhibits parasitism (Alakonya et al., 2012; Shen et al., 2020). Additionally, host-derived sRNAs (e.g., AtsRNA4348) may target genes such as leucine-rich repeat receptor kinases in dodder, affecting its growth or parasitic capacity (Subhankar et al., 2021). Host mRNAs can even be transmitted to feeding insects (e.g., aphids) through dodder, forming a unique three-kingdom RNA communication network involving plants, parasitic plants, and insects (Song et al., 2022).

sRNAs (including miRNAs) produced by dodder enter host cells and silence host immunity-related genes through RNA interference (RNAi), which represents a key strategy for its successful parasitism. For example, miRNAs generated by *Cuscuta campestris* (such as miR12463b and miR12495) target the 5′ and 3′ regions of *BIK1*, a critical factor in the Arabidopsis immune signaling pathway; miR12497a/b targets the auxin receptors *TIR1* and *AFB3* as well as the transcription factor *ABF2*; miR12494b targets *HSFB4/SCZ*; and other sRNAs target defense genes including *SERK2* and *SNAK2* (Shahid et al., 2018; Subhankar et al., 2021). Specific parasitic sRNAs (e.g., CcsRNA0641) can target host disease resistance protein family genes and sRNA-degrading enzyme genes, systemically suppressing the overall defense responses of the host (Subhankar et al., 2021).

Mobile RNAs from Cuscuta species reciprocally modulate host physiology to enhance stress tolerance and coordinate nutrient signaling. Cuscuta-transmitted RNA signals induce salt stress-responsive genes in hosts (e.g., citrus and cucumber), enhancing host salinity tolerance (Li et al., 2020; Duan et al., 2022). Transferred lncRNAs influence host genes associated with “metabolic processes,” “catalytic activity,” “signal transduction,” and “stress responses” (Wu et al., 2022). Specific sRNAs (e.g., CcsRNA4295) target host genes such as Golgi nucleotide sugar transporters, with their long-distance mobility potentially dependent on host factors like the Arabidopsis SGS3 protein (Subhankar et al., 2021)). Furthermore, Cuscuta-mediated mobile RNAs (including specific mRNA signals) transmit bidirectionally between hosts (e.g., among cucumber plants), coordinating resource allocation (e.g., nitrogen) (Zhang et al., 2021). Functioning as “messengers,” these mobile RNAs relay environmental stress information within the host–Cuscuta–host circuit (Duan et al., 2022; Wu et al., 2022).

Bidirectional mobile RNA trafficking occurs between parasitic plants and hosts, with subsets of sRNAs undergoing long-distance transport to distal organs (e.g., leaves and roots) to regulate stress responses, transmit physiological signals, or facilitate parasitism signaling (Shotton, 2018; Subhankar et al., 2021). Mobile RNAs act as central hubs in information exchange, spanning from host and parasite immune responses during parasitism establishment to post-attachment communication. From a systemic immunity perspective, defensive mobile sRNAs produced by the host plant traverse the haustorial interface, systemically suppressing growth of distal haustoria or nascent shoot apices beyond direct contact points, thereby establishing a non-contact immune barrier. Conversely, Cuscuta-derived immunosuppressive mobile RNAs diffuse within the host vasculature, systemically dampening host-wide defense responses to favor successful parasitism.

In development and nutrition, mobile RNAs ensure cross-species physiological synchronization and resource coordination. Bidirectionally trafficked nutrient-related mobile RNA signals (e.g., nitrogen-responsive mRNAs) precisely regulate photosynthetic assimilate synthesis in host source organs (leaves) and nutrient partitioning toward sink organs (roots and parasitic haustoria). This orchestrates a dynamic equilibrium between resource acquisition and host tolerance, underscoring the pivotal role of mobile RNAs in shaping the unique symbiotic–antagonistic relationship.

#### 2.2.4 Symbiotic plants

In symbiotic interactions, mobile RNAs act as key mediators of signal coordination between plants and their symbiotic partners. Symbiont-derived mobile sRNAs (e.g., tRFs and fungal sRNAs) diffuse through the vasculature, systemically suppressing defense gene expression and priming systemic immunity in non-contact host tissues. Conversely, mobile RNA signals synthesized in aerial plant parts traffic downward to roots, precisely modulating the rhizosphere microenvironment and the development of symbiotic structures (nodules, mycorrhizae).

In the rhizobium-legume symbiosis—crucial for nitrogen fixation and plant growth—*Bradyrhizobium japonicum* secretes transfer RNA-derived fragments (tRFs), primarily 21 nt in size, into soybean cells. These tRFs target soybean root hair development genes *RHD3a/b* and trichome meristem genes *HAM4a/b*, promoting nodule formation (Ren et al., 2019). Shoot-derived miR2111 moves downward via the phloem to roots, targeting the *TOO MUCH LOVE (TML)* gene. This interaction systemically guides lateral root initiation, nitrogen responsiveness, and rhizobial infection. Notably, this miR2111-*TML* regulatory module governing lateral roots is conserved even in non-symbiotic Arabidopsis, indicating a fundamental developmental function (Sexauer et al., 2023). Furthermore, rhizobial infection triggers extensive systemic RNA mobility. Numerous mRNAs (e.g., *SUCS1*, *DGD2*, *GS*, and *PUB1*) undergo long-distance transport between roots and shoots during early symbiosis, regulating nodule formation and nitrogen fixation (Zhang et al., 2020).

Beyond rhizobia, Silvestri et al. provided the first evidence of mobile RNA involvement in the symbiosis between plants and the AM fungus *Rhizophagus irregularis*. Fungal sRNA Rir2216 enters the host plant *Medicago truncatula*, targeting and downregulating the transcription factor gene *MtWRKY69*. This suppression of host defenses promotes fungal colonization and enhances symbiotic establishment (Silvestri et al., 2025). Whether plants reciprocally regulate symbiotic fungi by secreting sRNAs remains unconfirmed.

Through mobile RNAs, signals generated in shoots upon sensing nitrogen/phosphorus demands traffic downward to promote the formation and maintenance of root symbiotic structures while optimizing root architecture. Concurrently, information on root symbiotic status feeds back via upward-moving signals (e.g., *SUCS1* mRNA) to regulate shoot photosynthate synthesis and carbon allocation. This bidirectional long-distance communication underpins systemic symbiotic harmony by achieving cross-organ carbon–nitrogen/phosphorus balance.

## 3 Transport pathways for mobile RNAs

### 3.1 Extracellular vesicle-mediated transport

EV-mediated RNA transport is a core pathway in plant–microbe interactions. During pathogen infection, plants secrete EVs carrying sRNAs into invading microbes, disrupting their physiology through cross-kingdom delivery. In the cotton-*Verticillium dahliae* system, cotton-derived EVs are enriched with sRNAs like miR159 and miR166. These EVs fuse with the fungal plasma membrane, releasing miR159 and miR166 into the pathogen cell. These miRNAs precisely target fungal virulence genes (Zhang et al., 2016).

EV biogenesis is tightly linked to multivesicular bodies (MVBs). MVBs form through invagination, generating intraluminal vesicles that encapsulate cargoes like sRNAs. Subsequent MVB fusion with the plasma membrane releases these vesicles as EVs into the extracellular space (Moulin et al., 2023). EV lipid composition, particularly glycerophospholipid species and abundance, critically influences sRNA loading efficiency. In some plants, specific glycerophospholipids interact with sRNA-binding proteins, facilitating selective sRNA sorting into EVs for accurate delivery to target cells. Structural studies reveal that exosomal membrane lipid raft domains (enriched in sphingolipids and cholesterol) specifically bind AGO1-miRNA complexes, forming ~100-nm transport particles resistant to host extracellular nucleases (Song et al., 2021).

### 3.2 Non-vesicular transport

Non-vesicular RNA transport refers to the movement and extracellular presence of RNA species that are not packaged into extracellular vesicles (EVs) but instead exist as ribonucleoprotein (RNP) complexes or protein-associated RNAs in the cell wall/intercellular (apoplastic) space. Mechanistically, secretion into the apoplast can occur via multiple routes. Classical ER–Golgi secretion delivers signal-peptide-containing proteins to the cell exterior (Wang et al., 2017), whereas Type I unconventional protein secretion (UPS) pathways export leaderless proteins by MVB fusion, autophagosome/endosome-based routes, or other non-canonical translocation mechanisms (Maricchiolo et al., 2022). These UPS pathways provide plausible routes for RBP–RNA complexes to reach the apoplast without vesicle encapsulation, although the detailed molecular steps remain under active investigation.

Several RBPs have now been implicated in non-vesicular RNA transport (Borniego and Innes, 2023). In Arabidopsis, GRP7 protein binds specific circRNAs, protecting them from ribonuclease degradation. GRP7 may facilitate sRNA, lncRNA, and circRNA transport via non-vesicular secretion (e.g., direct binding followed by extracellular release) (Zand Karimi et al., 2022), and biochemical evidence demonstrates that the glycine-rich (GR) domain is important for protein mobility and for mediating sRNA cell-to-cell movement (Yan et al., 2020). As a conserved mechanism, distinct RBPs including SRBP1 and PSRP1 mediate non-vesicular transport of single-stranded sRNAs with length specificity, supporting intercellular and systemic sRNA signaling in plants (Yan and Ham, 2022). Additionally, AGO2 protein binds sRNAs and participates in non-vesicular transport. ago2 mutants exhibit heightened susceptibility to Cucumber mosaic virus (CMV) and Turnip crinkle virus (TCV), and AGO2 expression is induced upon infection, indicating its role in sRNA-mediated systemic RNAi defense against viral spread (Carbonell et al., 2012).

### 3.3 Vascular long-distance transport

The phloem serves as the primary conduit for long-distance RNA transport, mediated by ribonucleoprotein complexes (RNPs) formed with RNA-binding proteins (RBPs). During vascular transit, retention efficiency varies among siRNAs of different lengths (e.g., 21 vs. 24 nt). This selectivity is driven by tissue-specific expression of ARGONAUTE (AGO) proteins, where the presence of specific AGOs (e.g., AGO1, AGO2) determines siRNA stability, loading, and delivery (Devers et al., 2023). For instance, the PSRP1 (Phloem SMALL RNA BINDING PROTEIN 1) selectively binds 25-nt single-stranded RNAs in pumpkin, facilitating their transport through sieve tubes (Ham et al., 2014). Furthermore, tRNA-like sequences (TLS) and m5C RNA methylation enhance the systemic mobility and stability of mRNAs, respectively, promoting their accumulation and transport within the phloem (Zhang et al., 2016; Yang et al., 2019).

The directionality of mobile RNA transport is regulated by gene promoter activity and environmental stress (e.g., nutrient deficiency). Under sulfur or copper deficiency, miR395 and miR398 move via the phloem toward shoots, enhancing plant tolerance by modulating aerial gene expression (Deng et al., 2021). Conversely, *BEL5* RNA traffics to tuber-inducing sites under short-day conditions (Sharma et al., 2016). Recent work revealed a “hitchhiking” mechanism for mobile mRNAs: ROC family proteins, through coordinated action of endosomes and the cytoskeleton, precisely localize mRNAs to plasmodesmata, enabling continuous transport from local to distal sites (Luo et al., 2024). Remarkably, a single endogenous siRNA can regulate hundreds of distal transcripts across developmental and stress-response pathways, revealing broader endogenous functions than previously recognized (Devers et al., 2023).

### 3.4 Plasmodesmata-mediated intercellular trafficking in systemic RNA movement

Plasmodesmata (PD) are microscopic membrane-lined channels connecting adjacent plant cells, forming a symplastic continuum that allows direct cell-to-cell exchange of small molecules and macromolecules such as RNAs and proteins (Yan and Liu, 2020; Yan et al., 2020). While PD do not themselves mediate long-distance (systemic) transport, they play an essential role in local intercellular trafficking and in phloem loading/unloading, which are prerequisite steps for systemic RNA movement (Devers et al., 2023). Intracellular mobile RNAs utilize cytoskeleton-driven organelle transport for targeted delivery to PD. Mobile mRNAs (e.g., *KN1*, *FT*) are first recognized by specific RNA-binding proteins in the cytoplasm and assembled into RNPs. Recent studies show that RNA-binding cyclophilins (ROCs), acting as organelle-tethered RBPs, specifically bind mobile mRNAs. ROCs anchor mRNAs to Golgi or multivesicular body (MVB) surfaces, facilitating myosin-dependent trafficking along actin filaments and kinesin (e.g., KinG)-mediated transport along microtubules toward PD. Disruption of ROCs or the cytoskeleton (e.g., microtubule depolymerization) causes mRNA accumulation in the cytosol, preventing PD targeting (Luo et al., 2024).

Upon mRNA arrival at PD, pectin acetylation status—modulated by pectin acetylesterases—controls cell wall flexibility and PD permeability. In Arabidopsis, pectin deacetylation inhibits secondary cell wall (CW) extension, reducing secondary PD formation. This impedes symplastic movement of sRNAs (e.g., siRNAs and artificial miRNAs), GFP, and viruses between mesophyll cells. Notably, it does not affect transport in roots or via primary PD, highlighting the directionality and tissue specificity of RNA mobility (Jay et al., 2025).

## 4 Mobile RNA-based disease control strategies

Advances in understanding mobile RNA mechanisms in plant–pathogen interactions are driving the translation of theoretical discoveries into innovative disease control technologies. Engineered mobile RNAs and targeted delivery systems offer significant advantages in precision, systemic efficacy, and environmental compatibility, providing novel pathways for agricultural disease management.

### 4.1 Development and application of RNA interference biopesticides

The systemic mobility of RNAs provides a natural blueprint for RNAi biopesticides. Exogenously applied double-stranded RNA (dsRNA) or siRNA can induce endogenous plant RNAi pathways, triggering systemic defense responses against pests or pathogens. For example, spray-induced gene silencing (SIGS) of fungal dsRNA targeting essential genes (e.g., cytochrome *P450*) inhibits pathogen growth (Song et al., 2018; Islam and Sherif, 2020; Han et al., 2025). Similarly, dsRNA targeting plant viruses (e.g., Tomato yellow leaf curl virus) induces systemic silencing and reduces viral load (Holeva et al., 2021; Delgado-Martín et al., 2022; Konar et al., 2022). However, naked dsRNA/siRNA is susceptible to environmental nucleases and exhibits limited plant uptake (Romoli et al., 2024), severely constraining practical efficacy.

Nanomaterial carriers are increasingly employed to enhance dsRNA delivery. Systems like layered double hydroxide (LDH) and liposome complexes protect dsRNA and facilitate vascular transport, enabling control of pests and diseases throughout the plant (Koch et al., 2016; Jain et al., 2022; Cheng et al., 2024). LDH, an inorganic nanocarrier, adsorbs dsRNA via interlayer anion exchange, forming LDH/dsRNA nanocomplexes. This system confers nuclease resistance in tobacco, extending dsRNA half-life to 72 h—a threefold increase over free dsRNA (Cheng et al., 2024). Field trials demonstrated 82% suppression of whiteflies in LDH/dsRNA-treated tomatoes, with no significant toxicity to beneficial insects like bees (Jain et al., 2022). Huayu et al. complexed cationized bovine serum albumin (cBSA) with dsGUS RNA to form stable nucleic acid–protein nanocomplexes. In tobacco and poplar, localized application to petioles or shoot bases induced systemic gene silencing (Sun et al., 2024). Recent work utilized thiol-modified siRNA nanoparticles delivered via the vascular system to achieve long-distance gene silencing across monocots and dicots (Lin et al., 2025). These studies collectively demonstrate that nanocarrier-based delivery systems overcome the limitations of naked RNAs by effectively protecting nucleic acids, enhancing plant uptake, and leveraging vascular transport, providing key solutions for the practical implementation of RNAi biopesticides.

### 4.2 Gene editing and mobile RNA tools

**Figure 3:**
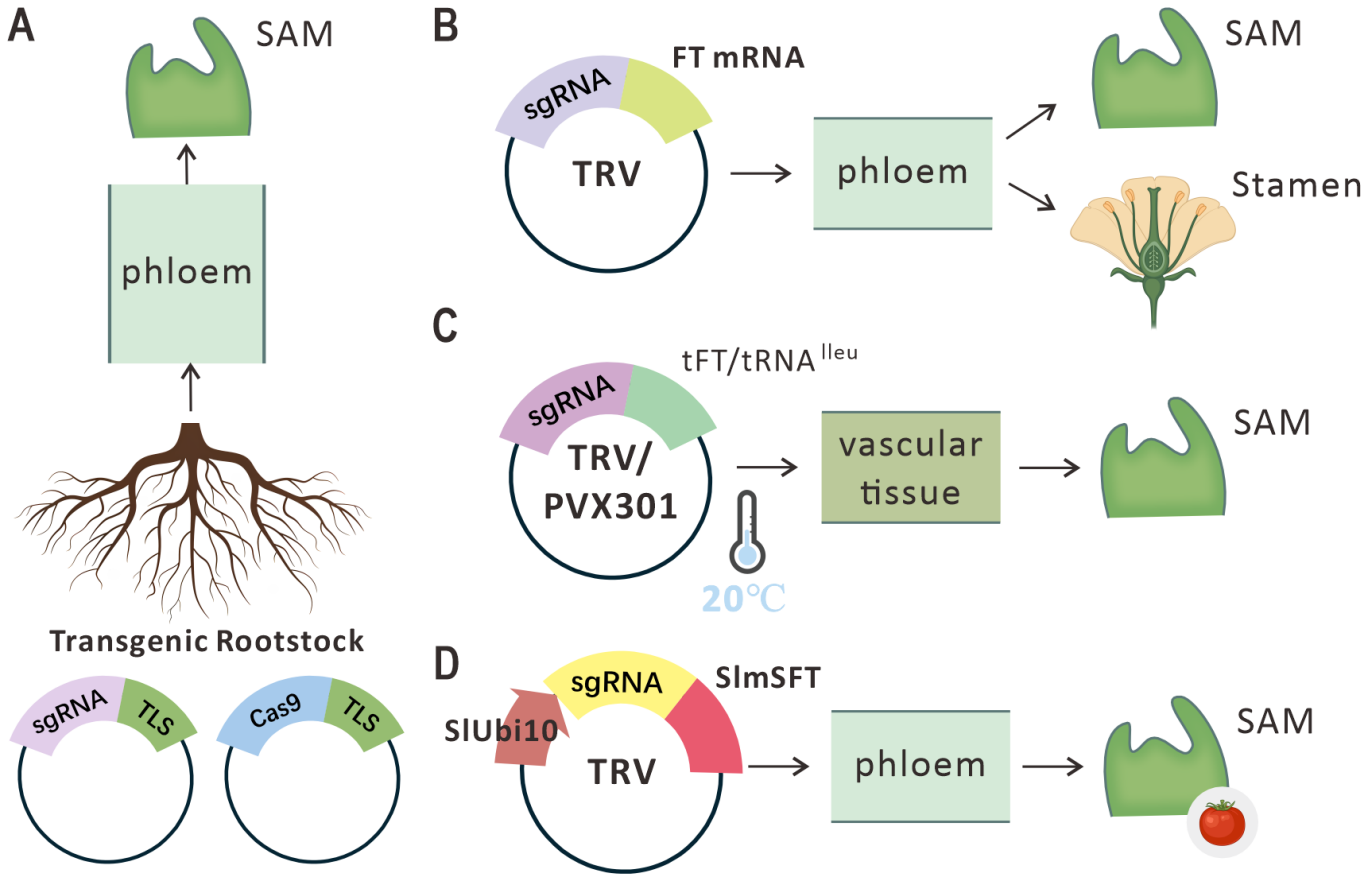
Mobile RNA-mediated delivery strategies for gene editing. **(A)** Fusion of sgRNA with endogenous tRNA-like sequences (TLS) enables formation of mobile ribonucleoprotein complexes (RNPs). These RNPs are transported from transgenic rootstock roots to the shoot apical meristem (SAM) of wild-type scions, achieving transgene-free gene editing. **(B)** Conjugation of sgRNA to Flowering Locus T (FT) mRNA sequences exploits FT’s inherent mobility for sgRNA transport. The infectivity of Tobacco Rattle Virus (TRV) further facilitates sgRNA dissemination to SAM and reproductive tissues. **(C)** Low temperature (20°C) suppresses plant antiviral defenses, enhancing TRV and Potato Virus X (PVX) accumulation to improve sgRNA delivery efficiency. **(D)** The tomato SlUbi10 promoter and SlmSFT exhibit enhanced compatibility with endogenous cellular transport mechanisms, promoting efficient sgRNA translocation to SAM.

Mobile RNAs demonstrate unique potential in plant gene editing. Their integration with editing tools overcomes limitations of traditional transformation methods, enabling more efficient, precise, and heritable genome modifications. By facilitating the systemic transport of sgRNA via sequence-specific elements, mobile RNAs enhance sgRNA delivery efficiency and range within the organism, bypassing tissue-targeting and efficiency constraints of conventional editing (**Figure 3**).

Studies show that fusing mobile RNA sequences (e.g., Flowering Locus T family mRNAs or tRNA-like structures) to sgRNA creates composite RNA molecules with enhanced mobility. These leverage plasmodesmata or the vasculature for long-distance transport of sgRNA from injection sites to key tissues like the shoot apical meristem (SAM) and germ cells (Kang et al., 2025). This transport enables sgRNA to precisely complex with Cas9 protein expressed intracellularly, directing DNA cleavage and inducing targeted mutations. Lei et al. achieved transgene-free heritable editing by grafting transgenic rootstocks (expressing Cas9 and tRNA-like-fused sgRNA) onto wild-type scions. In oilseed rape, tRNA-like sequences (e.g., AttRNAIle) promoted root-to-shoot sgRNA movement, inducing biallelic mutations in target genes (e.g., *NIA1*) with 0.45%–15.9% editing efficiency in progeny (Yang et al., 2023). Ji-Hui et al. exploited BYSMV (Barley yellow striate mosaic virus) to develop a negative-strand RNA viral vector system. Fusion of tRNA sequences enhanced RNA mobility, successfully delivering Cas9 and sgRNA to wheat meristems and overcoming delivery bottlenecks in monocots (Qiao et al., 2025).

Current approaches to boost mobile RNA-mediated sgRNA delivery encompass low-temperature modulation, promoter optimization, species-specific sequence adaptation, and viral vector integration. TRV, PVX (Potato virus X), and BSMV (Barley stripe mosaic virus) directly induce heritable mutations (TRV: 5.5%–15.4% editing efficiency), whereas FoMV (Foxtail mosaic virus) requires mobile signals (e.g., *ZCN16*) for somatic editing but fails in germline editing (Beernink et al., 2022). Owing to its SAM invasiveness, the bipartite TRV serves as an efficient delivery vehicle–TRV carrying-sgRNA generated 15%–100% homozygous mutant seeds directly in tomato fruit. Notably, RNA mobile signals alone are insufficient to achieve virus-mediated germline gene editing. The intrinsic properties of the virus—particularly its capacity to invade the shoot apical meristem (SAM)—constitute a critical determinant for successful germline editing. Critically, cold treatment enhances virus-induced genome editing (VIGE) efficiency by facilitating viral accumulation. In tomatoes, incubation at 20°C (vs. 25°C) suppresses host antiviral RNA silencing, prolonging the replication cycle of viral vectors TRV. This increases accumulation of sgRNA–mobile RNA complexes by 40%, elevating indel mutation rates from 12% to 68% (Kang et al., 2025).

Promoter engineering enhances editing efficiency by driving Cas9 expression in target tissues. The tomato SlUbi10 promoter outperforms the Arabidopsis AtUBI10 ortholog, increasing Cas9 activity 2–3-fold in shoot apical meristem (SAM) and germ cells. This achieved 70.9% target gene editing efficiency in transgenic line OX-3 (Lee et al., 2025). Species-specific sequence adaptation leverages endogenous host transport mechanisms. Fusion of sgRNA to tomato *SlFT* mobile sequences significantly enhanced transport efficiency over Arabidopsis *AtFT* homologs (54% nucleotide identity), improving sgRNA delivery 3-5-fold via specific interactions with tomato vascular RNA-binding proteins (Lee et al., 2025). Synergistic application of these strategies overcomes key sgRNA transport bottlenecks, enabling precise genetic improvement in crops.

## 5 Discussion

Recent studies employing dynamic detection techniques (e.g., TRAP-seq) suggest that short-read sequencing may underestimate the abundance of mobile mRNAs, necessitating integrated multiomics approaches to validate their transport potential (Paajanen et al., 2025). Future research should prioritize multiomics integration and interdisciplinary convergence. Combining single-cell transcriptomics (scRNA-seq) with spatial transcriptomics will map the spatiotemporal expression dynamics of mobile RNAs under diverse stresses, elucidating their regulatory networks in tissue-specific resilience responses. Systems biology approaches can model cross-stress response networks to identify key regulatory hubs mediated by mobile RNAs.

In biotic interactions, deciphering the exchange mechanisms of mobile RNAs between parasitic plants and hosts is crucial for discovering molecular targets applicable to biocontrol. From an evolutionary perspective, comparing mobile RNA sequences across plant species with divergent stress resilience (e.g., desert-adapted plants vs. crops) will reveal conserved functions and adaptive mutation patterns underlying environmental adaptation. For agriculture, mobile RNA technologies promise transformative “resilience-on-demand breeding”. Machine learning can predict target gene networks of mobile RNAs, enabling the design and deployment of “resilience modules” in crops—such as drought-inducible promoters driving mobile RNA expression—to create smart varieties responsive to environmental cues. Plant-derived EV-based RNAi biopesticides represent an emerging direction for ecofriendly crop protection; their inherent biocompatibility minimizes ecological risks. Coupled with synthetic biology, customizable EVs loaded with specific RNAs could enable precision management of pests and diseases. Furthermore, understanding how mobile RNAs orchestrate responses to combined stresses (e.g., heat, salinity)—exacerbated by climate change—is imperative.

Beyond viruses and endogenous RNAs, viroids offer a unique evolutionary perspective on RNA mobility. These small, circular, non-coding RNAs autonomously replicate and move systemically in plants, representing the simplest known form of mobile RNA pathogenicity. Recent studies show that viroid-derived small RNAs (vd-sRNAs) are processed by host silencing machinery and can guide RNA-directed DNA methylation (RdDM) and histone modifications, influencing host gene expression and development (Wassenegger and Dalakouras, 2021; Ortolá and Daròs, 2023). Importantly, their minimal RNA-only nature provides an ideal natural model to explore how RNA structure, sequence motifs, and mobility determinants coordinate cross-tissue transport and transcriptional silencing. Integrating these insights from viroid–host interactions with studies on viral and endogenous mobile RNAs will not only deepen our understanding of systemic RNA signaling evolution but also inspire the design of synthetic RNA systems for crop improvement (Flores et al., 2022).

Concerted multidisciplinary efforts will accelerate the green transition in agriculture: Materials science can develop stable RNA carriers; bioinformatics can enhance the prediction and dissection of mobile RNA regulatory networks; and agricultural engineering will bridge the gap from lab to field.
